# Analysis of Obligatory Involvement of Medical Students in Pandemic Response in the Czech Republic: Competencies, Experiences, and Legal Implications

**DOI:** 10.3389/ijph.2022.1605187

**Published:** 2022-12-22

**Authors:** Petr Michenka, Lydie Fialová, Lenka Šlegerová, David Marx

**Affiliations:** ^1^ Third Faculty of Medicine, Charles University, Prague, Czechia; ^2^ Independent Researcher, Aberfeldy, United Kingdom; ^3^ Institute of Economic Studies, Faculty of Social Sciences, Charles University, Prague, Czechia

**Keywords:** medical students, COVID-19, medical education, clinical competencies, Czech Republic, legal framework, healthcare capacity, crisis preparedness

## Abstract

**Objectives:** Medical students in the Czech Republic were mandated by the law to take part in the COVID-19 pandemic response in order to expand healthcare capacity. Our study aimed to analyze student’s competencies defined in the legislation and compare them with competencies assigned to them in clinical settings during their deployment.

**Methods:** Online survey with statistical analysis of collected data.

**Results:** The survey was completed by 997 respondents. A major convergence between the system of credentials defined in the legal framework and the competencies that students performed were identified.

**Conclusion:** Medical students represented a valuable resource for addressing shortages of qualified healthcare staff in critical situation. However, the system of competencies and credentials must be aligned with the educational framework to clearly define acquisition of competencies during the course of medical studies and the legal framework regulating students’ deployment must ensure consistency of actual and formal competencies in order to guarantee high standards of care and safety of the patients.

## Introduction

The pandemic situation represents serious strain on public health systems and requires increase in capacity as well as expansion of workforce in order to address healthcare needs of the population. In response to the COVID-19 (SARS-CoV2) pandemics, the discussion about the possibilities and limitations of employing medical students opened in many countries worldwide [1–4]. Although medical students had helped to tackle serious public health threats of the past, such as war or epidemics [5, 6], their role within healthcare systems in the times of crises and emergencies has rarely been addressed in practical, organizational, and legal terms as part of national emergency and pandemic preparedness plans. The aim of our study was to analyze the involvement of medical students in the pandemic response in the Czech Republic, focusing on the tension between the competencies and credentials of medical students and their assigned tasks in the clinical care during the pandemic response.

In the Czech Republic, the lack of qualified workers needed for effective pandemic response was most acutely experienced on the level of nursing care. This was caused by a combination of factors, such as increased hospital capacity demands, quarantine and isolation rules, government restrictions etc. [7]. Moreover, the altered spectrum of conditions of patients hospitalized during the pandemic—the provision of elective medical care was reduced and even canceled entirelym–have likely impacted the scope of competencies required to address patients’ needs.

To alleviate this problem the government declared the State of Emergency [8] which allowed for – and legitimized—the introduction of legally mandated “work obligation” for students of medical and nursing programmes [9]. In the history of the Czech Republic this was the first time the work obligation was applied selectively on one group of citizens for extended period of time. Previously, the work obligation was only activated during the massive flood events [10, 11].

**FIGURE 1:**
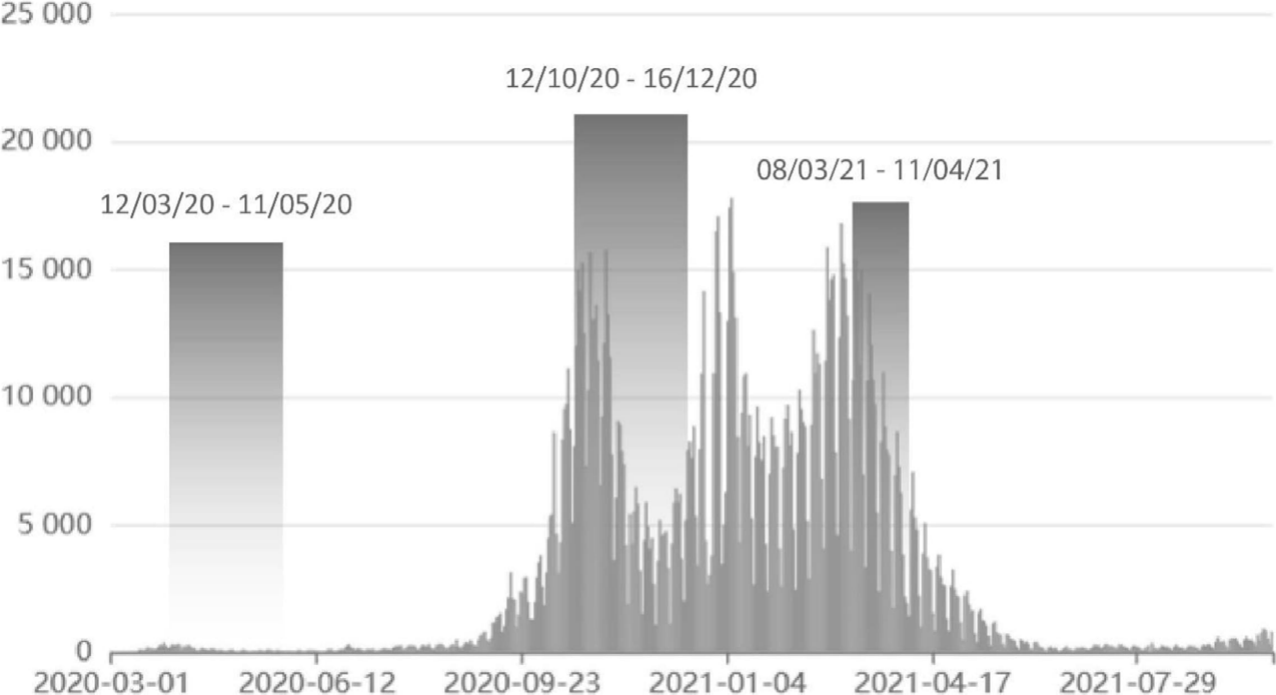
The timeline of COVID pandemic and the duration of the work obligation for medical students in the Czech Republic (Prague, Czechia. 2022, data: uzis.cz).

The purpose of this crisis management tool was to rapidly mobilize qualified citizens to serve public good. There was a strong correlation between the timing of the work obligation and the development of the pandemic in the Czech Republic, especially over the first year of the pandemic (March 2020–March 2021) (Figure 1). The involvement of students during other periods of the pandemic was on a voluntary basis. Intensity of this deployment varied based on regional needs during the pandemic.

While coping with increased numbers of patients, increased workload, limited resources, especially of nursing staff and creation of new working positions, healthcare facilities appreciated the help of medical students filling newly emerged workforce gap. According to the participating medical faculties, medical students were involved in the following areas:

• Standard healthcare positions—hospital orderly, medical care assistant, nursing aid
• Newly established healthcare positions—triage, swab testing, vaccination assistant, etc.
• Administrative positions—medical storekeeper, swab system management, helpline operator, etc.
• Supporting positions—babysitter for children of hospital staff

In this situation there was a hypothesis regarding a discrepancy between the formal credentialing system defined in the national legislation (in effect from 2004 to January 2021) and competencies assigned to medical students during their involvement in provision of care. Our study, unique in its scope and content, was primarily focused on gathering data on the forms and extent of involvement of medical students in clinical care during the pandemic response in 2020–2021, and on surveying both positive and negative effects of their involvement. Based on the results and areas of concern identified through our research, we discuss the potential and limitations of deploying medical students in crisis situations and on the practical, organizational, and legal implications of such involvement for healthcare system and medical education.

## The Organization of Medical Education and Credentials of Medical Students

Czech Republic is a Central European country with 10.702 942 inhabitants [12]. There are eight medical schools of four public and one state university, offering 6-year study programmes, and there are approximately 1865 graduates annually (i.e., 17.425 medical graduates per 100,000 inhabitants) [13]. The General Medicine programme is organized in preclinical section (years 1–3) and clinical section (years 4–6), with a minimum teaching time of 5500 h, awarding M.D. title (MUDr.) upon its completion.

Czech General Medicine curriculum is defined in the national legislation [14] and is in concordance with European framework [15]. It is accredited by the national authority, the National Accreditation Bureau for Higher Education [16], following standards of the European Association for Quality Assurance in Higher Education. The degree is recognized internationally, matching the European Qualifications Framework level 7 [17].

In order to qualify for any healthcare position, the acquisition of clinical skills and competencies must be confirmed by legally specified credentials issued by an educational institution [18]. Rather unusually in international comparison, medical students are endowed with credentials for two hospital positions in the course of their studies: Hospital Orderly (i.e., “Nursing aid”) after three successfully completed semesters; and Medical Care Assistant after four successfully completed semesters. Hospital Orderly performs auxiliary and service activities necessary for the provision of basic nursing, preventive, curative, and diagnostic care, mostly under supervision; while the Medical Care Assistants can also participate in basic nurse care provision independently and perform some procedures of advanced nursing care under supervision. This credentialing system was effective until 1. 1. 2021 when a new system was introduced in which medical students were granted credentials required to work as Practical Nurses after completion of eight semesters (120 weeks) of their medical study programme [19].

While this change reflected the shift in needs of healthcare to respond effectively to the pandemic during the data collection and the manuscript completion phase of our survey the executive orders necessary for its implementation were not finalized, and therefore this alteration was not reflected in our methodology.

## Methods

The national online survey KORONA 2020/21 was conducted from 23 March to 28 April 2021. The survey aimed at all students of the Czech medical programmes involved in the pandemic response studying the medicine excluding English speaking programmes. Seven out of eight medical schools in the Czech Republic participated and distributed the survey to their students enrolled in the General Medicine programme in the academic year 2020/21. For comprehensibility testing, the survey was first distributed to the students of the 5th year of the Third Faculty of Medicine Charles University. The subsequent full-scale distribution was conducted by the Study Departments of the participating medical schools *via* enclosed, login requiring university IT systems, using institutional encrypted email lists. All medical students received an explanatory email written by the authors, stating the purpose of the survey and information about the authors. To increase the response rate, students were also informed about the ongoing survey in Academic Senate [20] social media groups.

The survey consisted of 52 compulsory questions with additional voluntary comment questions, and was divided into three parts: 1. Demography (gender, medical school, study year, additional health-related education); 2. Student involvement (questions on the work obligation, number of shifts in different healthcare facility types, salaries, as well as the effects of their involvement in the pandemic response on their education); 3. Clinical competencies (drawing on their definitions in the legal framework). At the time of the survey conduction no surveys similar in focus nor methodology were available, thus, the validity testing was not performed.

For medical facilities involved in the pandemic response the following classification reflecting the assumed scope of the medical care provided was used in the survey: inpatient acute (hospitals); inpatient long-term (psychiatric, rehabilitation and geriatric hospitals); nursing homes and hospices; outpatient care (GP and outpatient specialists); and pre-hospital (EMS + triage). Only respondents working at least five shifts (i.e., “contacts” within the healthcare facility; at least 20 h of working) in any type of medical care were included in our competence analysis; and each student was included in only one category when working in multiple settings (in the category with the widest spectrum of care provided). Responses of students who did not participate in activities related to the COVID-19 pandemic and could not be included in any of the categories; students with additional healthcare education; and students with outlying answers for the question on the number of completed shifts (more than three standard deviations from the mean) were also excluded.

The clinical competencies part of the survey was based on the Czech legislation of 2004, covering competencies of healthcare workers in four distinct categories: “Hospital orderly,” “Medical care assistant,” “Practical nurse,” and “General nurse” [21], which reflect the gradual degrees of expertise and proficiency, independence of practice, and supervision required. In cases where a particular competency was included in more than one healthcare worker category, only the most detailed wording of the competency was used (Table 1). In some cases, competencies were subdivided because of the wide scope of competencies included. Three competencies were omitted because of they were neither specific enough nor relevant to the care provided. For the evaluation of the clinical competencies a scoring system was used: fifty-six selected competencies were grouped and scored based on the proficiency level of the healthcare worker category from whom the competency was adopted: competency of the “General nurse” was assigned four points, “Practical nurse” three points, “Medical care assistant” two points, “Hospital orderly” one point. Students who performed the given clinical competency (either with or without supervision) were granted number of points equal to the level of the competency. The sum of points equals the total value of competency level achieved by the individual student.

**TABLE 1:** Clinical competencies methodology, grey color: wording used (Prague, Czechia. 2022).

Hospital orderly	Medical care assistant
Assistance in feeding	Assistance in feeding and drinking
Measurement of patient’s temperature	Measurement of patient’s temperature, heart rate and pulse rate

**FIGURE 2:**
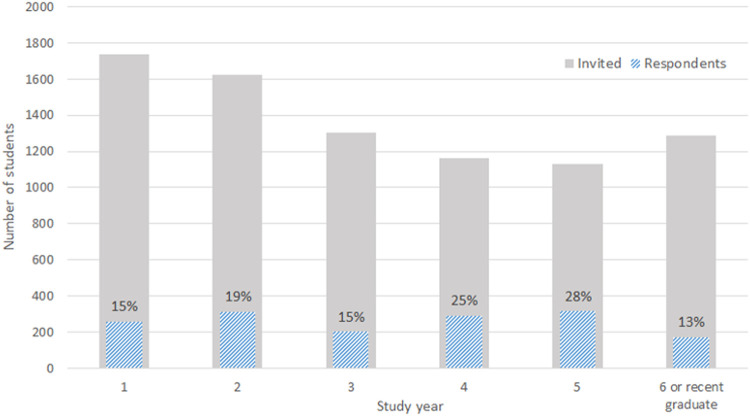
The real student distribution (grey) and the data distribution (blue) by the study years (source: Study departments of the participating medical faculties) (Prague, Czechia. 2022).

The distribution of the respondents among the medical faculties and study years was not balanced (Figure 2). Therefore, a sample balancing was used to weigh the data. Two sets of weights were assigned to each combination of the faculty and the study year. The first weighting set (balancing all the study years summed) was created by dividing the proportion of the respondents from each medical school and the study year by the real proportion of the medical students. This set was used to adjust the value of answers when the groups consisting of medical students from different study years were compared. The second weighting set (balancing each study year separately) reflected the disproportion between medical schools in the study years. This weighting set adjusted the answers when the variables across the study years were compared.

Differences among the groups were tested for several variables using the weighted Kruskal-Wallis test followed by the pairwise Wilcoxon rank-sum tests to identify the significantly different pairs. As the data shows non-normality, the Kruskal-Wallis test was used instead of the ANOVA test. The standard 5% significance level is used in reporting.

## Results

A total of 8,248 students were reached, out of which 1,550 returned the survey, 431 respondents were excluded from further analysis (students not involved during the pandemic). Furthermore, 89 respondents were excluded from the competency analysis as they did not work for at least five shifts in a healthcare facility; additional 17 were excluded as they had additional healthcare education and 16 provided outlying answers; resulting in the dataset of 997 valid responses. Although the exact number of Czech students involved during the COVID-19 pandemic varied, based on the available data of the governmental Medical Committee, 2,396 medical students were involved in the pandemic response [22]. This results in the response rate of 41.6% among deployed students. Response rates varied between the study years (ranging from 13% to 28%, Figure 2). The dataset consisted of 35% males, 64% females and <1% unspecified which corresponds with a normal medical student’s gender distribution in the Czech Republic [23, 24].

### Student Involvement

Students (*n* = 997) were often involved in more than one type of care setting at different times. The average number of shifts students worked varied across these settings: inpatient acute care (29.0; *n* = 710), inpatient long-term care (22.4; *n* = 112), nursing care homes (32.7; *n* = 39), outpatient care (26.5; *n* = 104), and pre-hospital care (32.6; *n* = 416). Within the category of inpatient acute care most of the students were involved in university hospitals (52.1%) and major regional hospitals (20.2%).

A negative effect of the involvement on the academic performance was reported mainly by 4th and 5th-year students, in 52% and 50%, respectively. The gain in theoretical knowledge was most positively reported by 1st-year students—67%, and a positive development in terms of clinical skills was mostly reported by 2nd-year students in 76% (Table 2).

**TABLE 2:** Effects of COVID involvement on medical students (Prague, Czechia. 2022).

Study year	Negative impact on study obligations (%)	Positive effect on theoretical knowledge (%)	Positive effect on clinical skills (%)
1	23.1	67.3	72.8
2	28.7	59.7	76.3
3	37.5	52.7	64.6
4	51.6	33.8	54.7
5	49.8	39.0	57.6
6	24.8	40.3	57.8

*Authors note: Respondents who reported “Definitely yes” and “Rather yes” included.

### Competency Analysis

On average, students (*n* = 997) achieved 60.6 competency points (CI: 54.3, 66.9), performed 20.5 competencies (CI: 18.4, 22.5) either independently or with supervision; with an average competency value of 2.96. The sum of competency points students achieved during their involvement did not constantly increase with the study years; however, an average value of the specific competencies did (Table 3). The mean value of the competency points was highest in the setting of acute inpatient care (73.6; *n* = 692), with the average number of the clinical competencies that students performed being 24.3.

**TABLE 3:** Competency points of medical students (Prague, Czechia. 2022).

	Competency points				The mean number of clinical competencies	The mean value of one competency
	Mean	25%	Median	75%		
Total (*n* = 997)	60.59	17.86	39.29	68.01	20.46	2.96
Study year						
1 (*n* = 43)	45.36	3.74	16.92	68.77	16.23	2.79
2 (*n* = 174)	58.21	15.75	38.14	73.52	19.90	2.92
3 (*n* = 123)	67.02	18.93	53.24	86.36	22.49	2.98
4 (*n* = 253)	68.14	35.15	70.04	97.54	22.75	2.99
5 (*n* = 280)	72.43	37.84	65.25	99.08	23.89	3.03
6 (*n* = 124)	60.43	10.69	44.59	88.43	19.72	3.06
Type of medical care						
Inpatient acute (*n* = 692)	73.57	30.51	48.73	78.28	24.26	3.03
Inpatient long-term (*n* = 65)	58.83	27.81	44.42	64.54	19.85	2.96
Pre-hospital (*n* = 175)	14.29	3.36	6.29	14.32	6.57	2.17

Both the competency points and the number of clinical competencies performed are highly correlated with the total sum of shifts covered (corr = 0.68 and 0.70, respectively), especially the number of shifts covered in inpatient acute care (corr = 0.70 and 0.70 respectively). In the acute care where the student exposure to nursing competencies was the highest, the following competencies were performed by more than half of the students (Table 4, *n* = 997), either independently or with supervision.

**TABLE 4:** Inpatient acute care: percentages of medical students performing clinical procedures with supervision or independently (quartiles differentiated) (Prague, Czechia. 2022).

Clinical competency	% Of student performing the clinical procedure independently or with the supervision in acute care facilities	Should be performed by
Perform comprehensive hygienic care, including prevention of bedsores	87.50%	Practical Nurse
Monitor, preliminary evaluate and record the physiological functions of patients using medical devices	86.20%	General Nurse
Distribute food to patients according to diets and ensure their adherence, supervise the adherence to the drinking regime, monitor fluid balance, take care of defecation	85.70%	Practical Nurse
Take care of the adjustment of the patient’s environment	85.60%	Practical Nurse
Administer medicinal products or medicines in the form of peroral administration	80.10%	General Nurse
Assist under direct guidance in designated nursing, diagnostic or therapeutic procedures	78.10%	Medical Assistant
Perform technical manipulations with beds, operating and examination tables and other medical devices	74.90%	General Nurse
Prepare medical material needed for the collection and further processing of biological material	73.80%	Practical Nurse
Administer medicinal products by infusion	73.60%	Medical Orderly
Carry out rehabilitative treatment in cooperation with the responsible person (positioning, sitting, basic passive, mobility training, ...)	72.10%	General Nurse
Provide activities related to the admission, transfer and discharge of patients	71.40%	General Nurse
Provide and perform examinations of biological material obtained by non-invasive means and capillary blood	70.60%	General Nurse
Introduce and maintain inhalation and oxygen therapy	70.60%	Medical Orderly
Administer medicinal products by sc injection	66.00%	General Nurse
Assist in the application of dressing materials, remove hard bandages from the patient	64.60%	General Nurse
Collect blood and other biological material and assess whether the results are physiological	64.10%	General Nurse
Evaluate and treat peripheral venous accesses, including ensuring their patency	63.70%	General Nurse
Dispose of biological material and contaminated consumables	63.10%	General Nurse
Care for established urinary catheters of patients of all ages, including ensuring their patency	60.90%	General Nurse
Perform treatment of uncomplicated chronic wounds	59.00%	General Nurse
Evaluate and treat skin integrity disorders	55.90%	General Nurse
To handle pressure vessels with medical gases	54.10%	General Nurse
Administer medicinal products by IV injection	51.00%	General Nurse
Perform transport, sorting and centrifugation of biological and medical material, distribution of laboratory in	50.90%	General Nurse
To serve food for special medical purposes	50.60%	General Nurse
Administer medicinal products by i.m. Injection	49.60%	General Nurse
Prepare specific dressing material as needed	47.60%	General Nurse
Introduce peripheral venous catheters in patients older than 3 years	42.10%	General Nurse
Assess and treat central venous accesses, including ensuring their patency	41.70%	General Nurse
Apply enteral nutrition to patients of all ages	41.40%	General Nurse
Provide and ensure psychological support to the dying and their loved ones and provide after the determination of death by a doctor	39.50%	General Nurse
Evaluate the needs and level of self-sufficiency of patients, manifestations of their illness, risk factors, even for	38.80%	General Nurse
Assist in initiating the administration of transfusion products and treat and terminate the patient during administration	37.60%	General Nurse
Acquire a personal, family, work and social history	37.20%	General Nurse
Perform treatment of surgical wounds	37.10%	General Nurse
Perform ancillary activities in the preparation of medicinal products, researchers and diagnostic medical devices	36.60%	General Nurse
Apply wraps, compresses, healing baths, hot and cold procedures	33.00%	Medical Orderly
To perform secretion of secretions from the upper respiratory tract in conscious adult patients and ensure their passage	32.70%	Medical Orderly
Perform treatment for drains and drainage systems	32.30%	Medical Orderly
Perform catheterization of the bladder of women and girls older than 3 years	32.00%	Medical Orderly
Perform treatment of acute wounds	31.70%	Medical Orderly
Perform stoma treatment	27.30%	Medical Orderly
Remove stitches in primarily healing wounds	26.50%	Medical Orderly
To perform secretion of secretions from the permanent tracheostomy cannula in patients older than 3 years and to ensure their p	24.70%	Medical Orderly
Check the temperature of refrigeration and freezing equipment	22.00%	Medical Assistant
Administer intravenous blood derivates	20.80%	General Nurse
Perform tracheostomy cannula replacement and treatment	20.10%	General Nurse
Introduce gastric tubes in adult patients	18.90%	General Nurse
Remove drains except for chest drains and head drains	14.70%	General Nurse
Performing a cleansing enema for patients older than 10 years	13.20%	General Nurse
Administer infusions or medicinal products in any form to a child under 3 years of age	9.80%	General Nurse
Performing bladder lavages	6.70%	General Nurse
To perform secretion of secretions from the upper respiratory tract in children and to ensure their patency	5.80%	General Nurse
Perform gastric lavage in patients with consciousness older than 10 years	4.60%	General Nurse
Administer medicinal products to an epidural catheter	4.00%	General Nurse
Perform skin treatment during radiotherapy	1.90%	General Nurse

Statistically significant differences in competency points exist both among the study years and the observed types of healthcare settings. Nevertheless, the differences are not significant for all possible combinations of the study years, i.e., 1st and 2nd study year (*p*-value = 0.083), 2nd and 3rd (*p* = 0.096), 2nd and 6th (*p* > 0.1), 3rd and 6th (*p* > 0.1), 4th and 5th (*p* > 0.1). There is also no significant difference between inpatient acute care and long-term care. For the number of competencies data entry, the insignificantly different pairs are the same as above with an additional pair of study years 1 and 6. For a robustness check, alternative sets of points were assigned to the General nurse, Practical nurse, Medical care assistant, Hospital orderly, i.e., 10-5-2-1 and 12-6-2-1, in contrast with the original 4-3-2-1 scheme. However, the statistical significance of the differences was not affected by this change.

### International Context

In order to contextualize our findings, we conducted preliminary international background research (October 2020 to February 2021) with an aim to gather information on work obligation application and student involvement. Embassies and Ministries of Health of all European countries were contacted *via* emails with questions on credentials and student recruitment during COVID-19 (Supplementary Material S1) The research confirmed that no unified terminology, system of competency-based study outcomes, or credentialing for medical students exist in Europe in contrast to that existing for medical graduates [25, 26]. Detailed international analysis would highly extend focus of this article and it would be desirable to cover the area in further research [27].

## Discussion

Our data suggest the national legal competency framework incorrectly defined the actual competency potential of medical students at all levels of student’s progression through medical school. Due to the lack of the alignment of educational and legal framework, healthcare facilities assigned competencies to students unsystematically. In general, majority of the reported clinical competencies were formally part of the nursing care skillsets. Drawing on the data (Table 4) we conclude that clinical competencies performed were mostly competencies formally corresponding to the highest level, i.e., “Practical Nurse” and “General Nurse.” It is nevertheless likely that during the crisis scenario with insufficiency of properly qualified workers, the required induction and adaptation process, lasting most commonly 3 months, was not adhered to suggesting students were not properly trained.

Using our methodology, the strong correlation between the study year and competency points can only be observed in the first three years of study years. Once in the clinical phase of the medical training the differences are statistically insignificant (Table 3). The most positive effect on clinical skills development was reported by the second-year students. This is understandable since the second-year students are already after their first contact with healthcare environment but did not have many opportunities to learn yet (more above). Thus, for them, the involvement during the pandemic facilitated and accelerated the clinical skill learning process the most. Interestingly, students of the final year reported a decrease in the absolute number of the competency points, which can be explained by several factors. Final year students had more study-related responsibilities (obligatory state exams) than the junior students and were exempted from work obligation in the 2020/21 academic year; consequently, the number of shifts staffed was lower. Also, a higher proportion of these students were involved in pre-hospital care with a relatively narrow spectrum of the care provided. However, it is important to highlight that both the sum of competency points and the spectrum of competencies reported by the final year students are still highly exceeding the official clinical competency framework. In terms of implication of the work obligation for the students it is nevertheless important to consider that more senior students’ years with the longest duration of the work obligation (4th and 5th study year) reported the most negative effects of the involvement on their studies. On the other hand, the reported positive effects are undeniable (Table 2). It is therefore highly desirable that the relevant authorities consider effects of these legal measures on medical education in greater detail—most importantly with emphasis on exposition of the students to clinical practice and the acquisition of competencies and skills [28–30]. Some students reported performance of competencies which would under normal conditions be exclusively assigned only to properly educated nursing staff (Table 4). This finding is further pronounced in the clinical study years (5th and 6th), where students could be profoundly involved in provision of healthcare. In some countries [31–34], a medical student with sufficient training can achieve a level of competencies nearly equivalent to the medical graduate working under supervision (e.g., “provisional doctor”). Therefore, an informed discussion with subsequent alignment of educational and legal framework should follow on a national level.

From crisis management point of view, in situations where an increased demand for healthcare workers is expected—especially over a longer duration—the capacity of potential workforce should be evaluated in advance [35]. This dimension of pandemic preparedness was not addressed sufficiently in the Czech Republic national pandemic response plans. Based on our analysis we recommend that instead of the year-based system of credentials currently used in the Czech legislation, a competency-based system aligned with the educational framework of study outcomes is more appropriate for assigning clinical competencies [36]. From our data, we conclude that the deployment of medical students has a strong potential to address shortages of qualified staff in critical situations in healthcare [37]. However, the legal framework for student’s deployment must be adjusted to reflect current medical educational frameworks of gradually acquired competencies and clinical skills. Vice versa, medical programmes should have official competency acquisition system reflected in the national legislation [38]. Additionally, medical education should reflect on the potential needs of healthcare system in critical situations and take them into consideration in curriculum design and development, to ensure relevant skills and competencies are acquired in a clearly defined and timely manner [39]. These adjustments have the potential to create more relevant, flexible, and effective system for assigning clinical competencies and improve crisis preparedness, while ensuring patient safety and quality of care. The probability of another large-scale pandemic is likely to increase in the upcoming decades [40], as is the likelihood of various health emergencies related to climate change, and the need for medical student involvement might reemerge [41].

Although introduction of the work obligation for medical students was a unique feature of pandemic response in the Czech Republic, the idea of students’ voluntary involvement during crisis situations periodically resonates abroad [42–45]. Available studies mostly focus on the sole potential of the students through mapping their willingness of being involved or general effects of the crisis situations on medical education [46–50]. Even recently, in Ukraine war, the government decided not to recruit full-time students without previous military experience for the service [51] and no data are available. Thus, relevant statistic, clinical or operational numbers or guidelines on potential involvement of medical students in healthcare provision are non-existent. Therefore, it would be highly desirable to focus efforts on these areas at domestic level and to implement necessary changes in national medical curricula and legislation.

From a wider perspective comprehensive international system of competency-based study outcomes in medical education would be beneficial. Currently complicated by differences in national legislations, medical curricula governance and non-uniform nomenclature [52, 53], if established, it could serve as an important factor for crisis preparedness and response effectiveness. This system would also facilitate student mobility, widen the opportunities for international collaboration in critical situation, as well as serve as a stimulus for modernization of medical school curricula to adapt to the evolving needs of population in health emergencies. International coordination of educational outcomes and legal frameworks governing medical competencies has the potential to impact healthcare provision in a global context and improve the preparedness for various crises in the future. Given its international significance, we call for a detailed comparative analysis of various national systems of clinical competences in medical education as a first step towards an informed debate about the benefits, potential risk factors, as well as ethical aspects of the deployment of medical student in healthcare [54].

Experience from the Czech Republic shows that medical students helped to alleviate the healthcare staff shortage and proved that the whole medical student community can significantly contribute to strengthen the healthcare system [55]. However, our analysis of their involvement uncovered an apparent convergence between the rigid competency system of credentials and the actual competencies and procedures that students performed during deployment in healthcare facilities. In conclusion, good clinical practice rests on evidence-based medicine. The same should be applied for setting of the proper inter/national legislation delineating medical students’ competencies, extent of their involvement in healthcare processes or their role in crisis situations. It is apparent that looking for an optimal solution will require additional data research, nevertheless, valuable lessons learned from the COVID pandemic can facilitate and inspire changes in processes to improve student experience [56], increase patients safety [57] and resilience of the healthcare systems [54].

### Limitations

The value of the findings is limited by the methodology used. The situation in the Czech Republic was unique in both extent of students’ involvement and competencies granted to them. Since students operated in many different healthcare facilities it was not possible to interview supervising staff and instead, students were surveyed. A potential bias of students’ self-evaluation [58] was mitigated in survey design using quantitative reporting instead of qualitative reporting. Survey response rate is calculated using limited governmental sources. From the beginning, the survey’s target group were only the students who were involved in provision of the healthcare during the COVID-19 pandemic, thus, students not involved were not included in the analysis. Due to the high number of questions used in the survey, we decided not to measure how many times a single competency was performed, and the competencies are reported in “Performed/Not performed” manner rather than in three more detailed categories (“Performed independently/Performed under the supervision/Not performed”) which would have provided additional information on functioning of the adaptation process and assignment of the competencies on-site, but inconveniently prolonged the survey. A compromise was also made during the selection of the competencies from the legislation. It is possible that if clinical competencies of only nursing positions (General and Practical nurse) were analyzed, more accurate results on the “out of the legislation scope” would be achieved. Finally, for a competency sub-analysis, a spectrum of the care was used as a primary classification parameter instead of the time factor (i.e., the number of the shifts). This assumed that the scope of clinical competencies is directly related to the spectrum of the care provided in given healthcare facility and not to the time factor. Despise these limitations, the presented limits, we believe that the methodology allowed to accurately analyze the practical implications of the current legislation.

## Supplementary Material

Supplementary Material S1 Collected answers of the international research (Prague, Czechia. 2022).
